# Equity, Health, and Sustainability with PROVE: The Evaluation of a Portuguese Program for a Short Distance Supply Chain of Fruits and Vegetables

**DOI:** 10.3390/ijerph16245083

**Published:** 2019-12-12

**Authors:** Daniela Craveiro, Sibila Marques, Ana Marreiros, Ruth Bell, Matluba Khan, Cristina Godinho, Sonia Quiroga, Cristina Suárez

**Affiliations:** 1 Instituto Universitário de Lisboa (ISCTE-IUL), CIS-IUL, 1649-026 Lisboa, Portugal; sibila.marques@iscte-iul.pt (S.M.); ana_catarina_marreiros@iscte-iul.pt (A.M.); cristina_isabel_godinho@iscte-iul.pt (C.G.); 2 Institute of Health Equity, UCL, London WC1E 7HB, UK; r.bell@ucl.ac.uk (R.B.); matluba.khan@ucl.ac.uk (M.K.); 3 Department of Economics, Universidad de Alcalá, 28801 Alcalá, Spain; sonia.quiroga@uah.es (S.Q.); cristina.suarez@uah.es (C.S.)

**Keywords:** short distance supply chain, fruits and vegetables, farmers, consumers, empowerment, health, equity, sustainability

## Abstract

PROVE is a Portuguese program that empowers small-scale farmers organized into local networks to directly commercialize baskets of locally produced fruits and vegetables to consumers. This study applied a post-test-only non-equivalent group design to evaluate the resulting influence on the social empowerment of farmers and on consumer diets. The method included conducting a survey of PROVE farmers (*n* = 36) and a survey of PROVE consumers (*n* = 294) that were compared against matched samples of Portuguese respondents of international surveys (European Social Survey, *n* = 36 and the INHERIT Five-Country Survey, *n* = 571, respectively). PROVE farmers reported higher scores for perceived influence on the work environment than the national sample. PROVE consumers were more likely to eat five or more portions of fruits and vegetables a day in comparison to the matched sample of Portuguese citizens (average odds ratio: 3.05, *p* < 0.05). Being a PROVE consumer also generated an impact on the likelihood of consuming no more than two portions of red meat a week (average odds ratio: 1.56, *p* < 0.05). The evaluation study suggests that the promotion of short supply chains of fruits and vegetables can make a positive contribution to a healthier, more sustainable, and fairer future in food consumption.

## 1. Introduction

Our food production system contributes around a third of the greenhouse gas emissions deriving from human activities and hence is interconnected with global warming, climate change, and the degradation of ecosystems that directly and indirectly impact on the general health and well-being of populations (e.g., [1,2]). The prevailing model of food production depends on industrialized and large-scale agricultural production and the implementation of long-distance supply chains [2,3]. Food transport distances and the number of intermediaries between producers and consumers interact with the environmental costs stemming from transportation, package, conservation, and food waste. Additionally, this kind of system produces sharp imbalances among the actors involved, privileging retailers over farmers and alienating consumers from food production processes [3]. Promoting short food supply chains has been proposed as a valuable strategy for counteracting some of these trends and promoting a fairer and more sustainable food production system [4,5,6]. This involves ensuring closer links between consumers and producers and decreasing the distances between the sites of food production and those of its consumption. Nowadays, a wide set of tools, regulations, and subsidized programs are in place to foster shorter food chains in Europe reflecting their feasible contributions towards sustainable agriculture [5].

Nevertheless, researchers note that shorter food circuits do not per se ensure advantages in terms of environmental costs. Contrary to some perceptions and policy discourses, locally produced food does not always represent the most environmentally friendly option and close empirical assessments are correspondingly necessary (e.g., [7,8]). Although there is increasing interest in short food supply chains in policy and research domains, most studies adopt a descriptive case study approach, with only a few studies evaluating the effects of the food supply chain on farmer and consumer perceptions, behaviors or the circumstances with experimental or quasi-experimental designs [4,9,10,11,12,13,14,15,16,17]. More comparative studies are needed to estimate the effects of such initiatives, but there are many technical difficulties in assessing the effects of ongoing practices and robust evaluation studies are too costly in terms of time and resources. 

In the literature reviewed, we found that articles focused mostly on assessments of localized programs such as farmers’ markets, cooperatives or selling schemes [9,10,11,12,13,14,15,16,17]. These publications tend to describe the initiatives and address impacts based on the perceptions of the experience of their users, collected by interviews and surveys. The trend illustrates time and resources constraints in implementing evaluation studies of ongoing projects. Much less often, some papers refer to the implementation of policies or broad socio-educative projects [18,19,20,21]. Among these cases, more solid methodological approaches are presented, supported by analysis of primary and secondary data and comparative studies. The literature describes experiences of short food chain initiatives implemented in African, American, and European contexts. Overall, the research findings suggest that the benefits of programs promoting local consumption are context specific and dependent on the features of both producers and consumers.

Studies on short food supply chains report benefits for both farmers and consumers. From the perspective of farmers, these studies highlight the role of these supply chains in fostering economic benefits, for example by ensuring access to new markets, opportunities to cut costs, and optimize the farming business related transport and logistics [4,9,10,11,12,13,14,15,16,17]. By securing the contexts of collective influence, programs promoting short food supply chains emerge as valuable strategies for enhancing the empowerment of farmers, increasing their capabilities for collective influence and decision-making in their farming activities (e.g., [11,13,22,23]). For example, Schimdt and collaborators [15] reported how a community-supported agriculture program in Vermont benefits farmers by increasing access to new individual and institutional consumers, such as businesses, schools, and restaurants, enabling them to increase sales. Kingu and Ndiege [11] reported the experiences of two dairy co-operatives from the Hai District in Kilimanjaro in optimizing farming and commercial strategies and thereby economically empowering small-scale farmers. Mancini and collaborators [13] also focused on dairy farmers but from the Parmigiano region in Italy, and reported on the perceived positive social, environmental, and economic impacts. The study points out how the program especially benefited small-scale farmers, ensuring conditions to increase sales and promote self-esteem.

Farmers economic empowerment was also reported in the assessment of national and regional policies that included the promotion of short food distribution chains. Assis and collaborators [4] presented a systematic review on studies concerning the impact of a Brazilian governmental policy, the PAA (Food Acquisition Program), implemented in 2003, as part of the national strategy to end hunger. The program promotes local small-scale farming and the governmental acquisition of food from local farmers to supply the need of social organizations. The seven studies indicated associations of the policy with improving social conditions and the income of local small-scale farmers, and improvements in food security among socially vulnerable families. The authors state, however, that the quality of the studies did not allow causal connections with the program. 

In the American context, Pitts and collaborators [20] presented a preliminary assessment of the Ten North Carolina CTG Project designed to implement and foster farmers’ markets in the region. The study targeted rural residents and was based on a randomized survey in three counties, collected at two points in time, in 2013 and 2014. Improvement in market use was only found in one of the evaluated settings, demonstrating the importance of contextual features in shaping public policy efficiency. 

Also, in the United States, Oberholtzen and collaborators [19] reported an assessment of the impact of Federal Nutrition Benefits on the farmers’ markets, based on data collected from 16 states in 2010. The study reported an increase in market use and highlighted the point that farmers that sell fruits and vegetables and are more dependent on markets were the ones more likely to perceive economic benefits from federal programs. 

More comprehensive accounts were made in the assessment of interventions combining social and educational elements targeting small-scale farmers’ families in two African countries, one in Malawi [18] and the other in Liberia [21]. Both projects intended to contribute to children’s health and well-being by promoting the farming business of local farmers, and the evaluation studies used pre- and post-comparative studies with matched control groups. 

Kerr and co-authors [18] reported on the effects of the participatory agricultural program targeting families living in the northern Malawi region, that included interventions in farming practices and family nutrition behavior. The research collected longitudinal data on children under the age of three years in nine periods across six years (2001 and 2007) in treatment and control groups. The study reported improvement in the nutritional state of children, only when considering participants involvement over time and not by simply comparing treatment and control groups, referring to the importance of following projects across time to understand their effects. 

Elsewhere, Rutherford and collaborators [21] studied the Agriculture for Children’s project, a rural agricultural project in Liberia. The authors found evidence for the modernization of farming practices and improvement of food security among farmers’ families, yet no significant advantages for the program participants in terms of the improvement of children outcomes in nutrition, health, and education.

From the consumers’ perspectives, the gains stem from access to fresh, high-quality food and closer connections with the farmers [9,10,15,16,24,25,26]. Perspectives from consumers attending three farmers’ markets in the Niagara region (Ontario, Canada) were collected in 1999 and highlight the importance from the consumers’ perspective of accessing high quality and fresh products and of supporting local farmers [10]. Supporting local farmers is also a main motive for the participants in food initiatives in two Canadian cities (The Good Food Box and New City Market Local Food Hub), who were also found to be very satisfied by the quality and price of local products [9]. Quality and price were found to be key concerns of customers at farmer markets in Stockholm (Sweden), while customers were less concerned about farmers and production conditions [16]. Additionally, research suggests that these closer links to famers promote healthier diet options, such as the highest intakes of fresh, seasonal products [10,27,28,29,30]. Studies on consumers’ perceptions advocate for these connections. For example, a study of the perceptions of change of eating habits among farmers’ markets customers in Italy suggested that the continued use of farmers markets can contribute to shaping food habits, namely increasing the consumption of vegetables and organic products [29]. Another study that collected perceptions from residents from two low-income communities in Chicago indicated considerably more access to fresh fruits and vegetables in farmers markets than in other types of outlets [17]. More robustly, the connection is demonstrated as a negative correlation between farmers market density (number of farmers markets by area) and body mass index among Italian adults, suggesting that direct commercialization between farmers and consumers may promote healthier diets [29]. This links shorter food circuits with diet-related health and sustainability benefits due to the connection between diets, health, and the environment.

There is convincing evidence that relates higher intakes of fruits and vegetables with better health, fewer chronic conditions, and lower risks of non-communicable diseases, such as cardiovascular diseases and several types of cancer [1]. Additionally, fruits and vegetable intake is an important component for environmentally sustainable dietary patterns given that replacing calories sourced from meat or animal products with plant-based foods decreases the environmental footprint of diets [30]. 

This study presents an evaluation of PROVE, a Portuguese program promoting direct links between consumers and agricultural product growers. PROVE was identified as a promising practice in the consumption domain by the European Union (EU) Horizon 2020 funded INHERIT project—INHERIT (INter-sectoral Health and Environment Research for InnovaTion)—in view of its plausible contributions to health, environment, and equality in the field of consumption [31]. The study applies the INHERIT model [32], a relational model that integrates the DPSEEA (drivers, pressure, state, exposure, effect, actions) model with the behavior change theory and takes into account distributional effects that contribute to inequities. Applying this theoretical framework to PROVE, in this study we hypothesized that being a PROVE customer is associated with higher consumption of locally grown fruits and vegetables than is typical in Portugal, and that PROVE empowers farmers and improves their material living conditions. In the scope of the INHERIT, an intervention is considered as contributing to health if it improves the chances of people performing behaviors with known connection with better health; as contributing to environmental sustainability if it improve the chances of people changing behaviors with relative lower environmental costs; and contributing to equity if targeting lower socioeconomic populations or addressing barriers that penalize options in vulnerable populations and are a disadvantage for health and well-being [33].

This paper introduces the study of the effects of the ongoing PROVE program on Portuguese farmers and consumers. The paper aims to assess using empirical evidence (1) on the connection between the PROVE program and empowerment of small-scale farmers in Portugal; and (2) the connection between PROVE and healthier and more sustainable diet options. 

It constitutes an original contribution to the literature by addressing the effects of PROVE, a Portuguese short food supply chains project not reported in the literature. To the authors’ knowledge, this is the first time that an evaluation of this type has been reported in the context of research on short food supply chains. The paper proposes a valid research strategy resorting to the comparison between users and matched samples of secondary data. 

PROVE was launched by ADREPES (The Association for the Rural Development of the Setúbal Peninsula) under an EQUAL Community Initiative and incorporated three stages (needs assessment, development, dissemination) consolidated under the scope of PRODER (the Rural Development Program 2007–2013). PROVE has now grown into a nationwide program, comprising of 120 small scale-farmers organized into 108 local groups across the country and with more than 4000 consumers committed to its principles of more sustainable production and consumption. The multiple local units act interdependently. The PROVE website aggregates all these local groups and provides indicators related to the functioning of their projects while also enabling direct contact between consumers and farmers. 

PROVE may be understood either as a toolkit or as a methodology developed to empower small-scale farmers to build their businesses based on selling directly to consumers (PROVE groups). This involves three types of actors: promotors, farmers, and consumers. The promotor is the entity or group of citizens that triggers the implementation of a new group. They reach out to PROVE territory partners in order to access the training needed to advance with implementation. The set of PROVE rules and best practices are presented to farmers at training sessions. All the steps involved in implementation are defined and explained in a handbook—covering everything from planning the farming to the commercialization phases. Farmers also gain access to an online platform connecting consumers and producers, which links to the national PROVE webpage. PROVE consumers subscribe to receiving baskets of an agreed range of fruits and vegetables with average weight of 7 kg. PROVE is a short circuit chain: there are no intermediaries between consumers and producers and the distance from ‘farm to plate’ is less than 50 km.

## 2. Materials and Methods 

### 2.1. Research Design

The evaluation design was theory-driven, based on the INHERIT Common Analytical Framework, therefore implying an intersectoral perspective spanning the health, environment, and equity implications of PROVE [32,33]. Not discarding the plural interplay between the PROVE program and the dimensions of equity, health, and environmental sustainability, the quantitative studies approached the contribution PROVE makes to both empowering farmers and influencing consumer diets. The theoretical relevance and availability of data together determined this evaluation focus. 

Two data collections were conducted within the scope of this evaluation: a phone survey targeting PROVE farmers (carried out in October 2018) and an online survey targeting PROVE consumers (taking place in November 2017–January 2018). Additionally, the PROVE evaluation study extended to include qualitative perspectives on the perceived results collected via a focus group including key project stakeholders. The presentation of this qualitative study falls beyond the scope of this article but can be consulted elsewhere [33]. 

As the PROVE program is well established, it did not allow for the randomization of control and intervention groups. Therefore, the evaluation assessment was based on the comparison between PROVE users and matched samples of national representative surveys (INHERIT Five-Country Survey, European Social Survey), following a “post-test-only non-equivalent group design” [34]. 

Following the procedure explained by Randolph and co-authors [35], to reduce selection bias from the collected samples, PROVE farmers and consumers are compared to samples with similar socioeconomic features applying a propensity score matching procedure—“a statistical technique in which a treatment case is matched with one or more control cases based on each case’s propensity score” ([35], p. 1). The procedure was applied to create data sets with matched cases to perform comparative studies between PROVE and non-PROVE users. 

### 2.2. Study Participants 

#### 2.2.1. Farmer Study

Phone interviews were conducted using the list of contacts publicly available: from 120 listed participants only 69 active phone contacts were available (there is often only one phone contact available per local group). The initial contact was made by text messaging, including a brief presentation of the project and survey. 

Thirty-six PROVE farmers participated in the study, corresponding to 54% of the listed farmers and 32% of the PROVE farmer universe. Their ages varied between 26 and 75 (mean (M) = 44.5, standard deviation (SD) = 10.7), 15 were male and 21 were female. Participants were distributed by education groups, with higher concentrations in the lower and higher levels: primary/lower secondary (36%), upper secondary (16.7%), and tertiary (47.2%). Most participants came from the North (44.4%) and Lisbon regions (38.9) with their agricultural activities tending to be ensured by family or mostly family work (78%). 

In the sample, the farmed areas varied from 0.3 ha to 12 ha with over 50% reporting a farmed area of 1 ha or less. PROVE farmer groups comprise of between 1 and 10 farmers even though most are quite small (with 70% composed of 1 or 2 farmers). Participants also varied in terms of their length of enrollment in PROVE. The great majority of participants (32 out of 36 interviewed farmers) stated that PROVE improved their material living conditions. On average, the reported duration of PROVE membership stands at around 6 years (average: 6.6 years, SD = 3.5), but with over two thirds of the sample having participated in the PROVE program for 5 or more years (77%). 

These farmers were compared with a subsample of the Portuguese sample from the eighth wave of the European Social Survey (ESS8) in the evaluation study. The ESS includes measures of attitudes, beliefs, and behaviors across European countries. For this specific study, we selected variables for sociodemographic information and personal empowerment (perceived influence in the work environment). 

The information provided in the survey did not allow for restricting the selection to farmers. To ensure comparability between the samples, income served as one of the matching variables. The next step involved selecting a subsample of the Portuguese ESS8 sample with similar features of the farmers PROVE sample, based on a propensity matching procedure [35]. This procedure received support from R software (Bell Laboratories (formerly AT&T), New Jersey, NJ, USA) and the MatchIt package [35] and Exact technique enabling the selection of a sample from the ESS8 with the same features as the PROVE sample in terms of gender, age group, education group, and household income. Many more cases could be selected from the ESS sample. We opted for a procedure to select a sample of similar size of the control group to not artificially inflate the sample effects significance with a bigger sample. Other strategies could be used. 

The PROVE farmers were compared with 36 matched cases from the ESS8: 15 men and 21 women, with their ages varying from 23 to 81 (M = 50, SD = 15.9), with a similar distribution of education (primary/lower secondary, 33.3%; upper secondary, 19.4%; tertiary, 47.2%) and region of origin variables (e.g., North, 50%; Lisbon, 33%).

#### 2.2.2. Consumer Study

The PROVE consumer questionnaire was drafted in close articulation with the Portuguese version of the INHERIT Five-Country Survey [36]. PROVE consumers were recruited by an online advertisement campaign that ran on PROVE communication channels (between July and November 2018). To ensure data quality, the study only considered individuals that completed the questionnaire and excluded responders both who took less than 40% of the median time for responding and those who took over three times the median response time. The selected sample was composed of 294 participants and thus corresponding to 6% of the known universe of active consumers in 2018 *(n* = 4875).

The PROVE consumer respondent ages varied between 22 and 73 (M = 44.5, SD = 10.7), were mostly females (80%), with colleges degrees (87%), living in urban regions (90%), and from Lisbon (60%) or the North region (34%). Around two thirds of the sample declare household incomes corresponding to the higher terciles of national income (>€1500/month) in Portugal. Participants reported being either fully employed (59%) or self-employed (35%). The survey reached consumers with varying lengths of adhesion to PROVE—from 12 years to just a few months. The average subscription time was 1.5 years (M = 2.5, SD = 2.4) even while half of the sample declared having held a subscription for over 2 years. Consumers tend to order a PROVE delivery either every two weeks (62%) or on a weekly basis (32%). 

The PROVE consumers were compared with a subsample from the INHERIT Five-Country Survey [36] for this evaluation research. The data collection for the INHERIT Five-Country Survey was carried out in the period from July to November 2018. After data screening procedures, we considered 1650 valid responses from Portuguese households. We selected a subsample of this survey based on the propensity matching procedure generated by R software and the MatchIt package [35] and the Coarsened Exact Matching technique (selected among the alternatives as it was the technique that returned better results in terms of reducing the propensity scores between samples). The variables considered in the matching procedure included education level, age group, gender, region, and size of place of residence (urban, rural). The procedure identified 1087 unmatched cases and 571 matched cases with the PROVE sample. 

The evaluation study compared the PROVE consumers sample with the 571 matched cases from the INHERIT survey. The selected sample from the INHERIT survey contained 55% of females, with their ages between 35 and 49 (48%), with higher education degrees (56%), and living in Lisbon (57%) or the North region (34%). 

### 2.3. Measures 

#### 2.3.1. Empowerment: Perceived Influence in the Work Environment 

The selection of empowerment indicators was constrained to the available information in the ESS questionnaire. In the consulted literature, farmers’ empowerment is typically assessed by indicators related to the implementation of farming practices, and economic well-being indicators, such as income, sales, and food security. Since this information was not available for comparison purposes, alternative measures were used based on a broad conceptualization of empowerment, based on a social psychology framework.

Two measures of perceived influence in the work environment were selected to assess the role of the PROVE project in the empowerment of farmers. According to the empowerment theory, the way people perceive their ability to influence social environments constitutes a key component of personal empowerment. This intrapersonal component relates to the interactional and behavioral components of the construct that enable people to influence people and actively participate in social and political contexts [37]. 

Within this scope, the assessment of intrapersonal empowerment encapsulates the perceived influence in work environments focusing on two particular dimensions. The first considers the perceived influence over their daily working activities. Participants thus answer, on a scale of 0 to 10, in which 0 = ‘I have/had no influence’ and 10 = ‘I have/had complete control’, how much the management of their work allows them to decide on the organization of their own daily work. The second reflects the perceived influence over organizational decision-making. On a scale of 0 to 10, in which 0 = ‘I have/had no influence’ and 10 = ‘I have/had complete control’, they indicate to what extent the management allows/allowed them to influence policy decisions about either the activities of the organization or the activities of their PROVE group, in INHERIT and PROVE samples, respectively.

#### 2.3.2. Diets: Healthy and Sustainable Options

To account for the role of PROVE in promoting healthier and more sustainable diets, this study takes into account the differences between the INHERIT Five-Country Survey and PROVE consumer survey samples concerning their intake of fruits and vegetables and red meat. This applied a standardized 15-item self-reported food frequency questionnaire (FFQ) to collect dietary information [38].

The World Health Organization (WHO) recommends the consumption of at least five portions a day of fruits and vegetables to decrease, among other health risks, the risk of serious cardiovascular diseases and some cancer types. To study whether PROVE consumers are more likely to maintain healthier diets than a matched sample of respondents in the Portuguese respondents of the INHERIT Five-Country Survey, we compared the samples in terms of their likelihood to follow this recommendation (hence, at least five portions a day or less than five portions a day). Considering the particularly high carbon footprint associated with red meat consumption, the PROVE implications for diet sustainability are assessed by comparing levels of red meat intake. Responders are compared regarding whether or not they consume less than two portions of red meat (200 g) a week, a recommendation in the Wheel of Five program from the Netherlands Nutrition Centre [39] that sets this limit in order to account for both health and sustainability reasons.

#### 2.3.3. Socioeconomic Variables 

Eight indicators acted as descriptors for the socioeconomic features defining the samples and are considered as control variables: gender, region, size of location (urban versus rural settling), age group (18–34 years old, 35–50 years old, 50+ years old), education group (primary/lower secondary, upper secondary, tertiary), household income (household terciles), perceived economic difficulties (no difficulties versus some economic difficulties), marital status (partner, no partner). Participants were also asked about the length of time of their PROVE participation. 

### 2.4. Data Analysis 

Comparative studies of samples adopted regression models for each dependent variable in which a sample identifier variable, PROVE (PROVE, non-PROVE), is introduced along with a set of controls. In order to handle any missing values, this process applied pairwise deletion procedures. In the consumer study, the model variables presented a rate of less than 2% of missing data with the exception of the gender variable in the consumer study. Due to the higher rate of missing cases (7%), this variable was coded as a three-level category: male, female, and no information. In the farmers study, only three cases included any missing values.

For the farmers study, two ordered regression models were conducted, since the scores for perceived influence in work environment were measured in a long Likert scale from 0 to 10.

For the consumers study, we modeled two logistic regressions to compare the likelihood of complying with diet recommendations among PROVE and non-PROVE consumers. The selection of control variables for introduction into the models arose from a pre-study concerning the INHERIT Five-Country Survey sample *(n* = 1658) in which the correlation between socioeconomic indicators and diet intake stemmed from unifactorial regression models (gender, age group, education group, partner, and economic difficulties were returned as statistically related to the intake of fruits and vegetables and/or red meat) [33].

The difference in the outcomes between PROVE and non-PROVE participants may not only be due to observable heterogeneity but also due to unobserved heterogeneity in our regression models [40]. To ensure that after the matching procedures, samples selection processes do not bias the results, we ran endogenous switching regressions (ESR) to account for endogeneity of the selection decision and tested the statistical adequacy of the resulting estimations. This exercise was done for the farmers study and consumers study and results are presented in the Supplementary Files. The bias treatment-effects test reported no evidence for sample selection bias.

## 3. Results

### 3.1. PROVE and the Empowerment of Small-Scale Farmers

#### 3.1.1. PROVE and the Perceived Influence in Daily Work Decisions

On average, PROVE farmers presented higher scores for the perception of influence over their daily working decisions (mean = 8.36, standard deviation = 1.46, *n* = 36) when compared with the non-PROVE sample (mean = 7.86, standard deviation = 2.17, *n* = 36). To compare the PROVE and non-PROVE samples, a set of ordinal regression models were estimated for these indicators, deemed as proxies for interpersonal empowerment. From the selected controls, only the education level presented a significant effect on the perceived influence in work environments. Participants in the lower education group score relatively lower than participants with tertiary education. 

Controlling for the effects of the age group, education group, gender, and economic difficulties, the sample effect, PROVE variable (PROVE versus non-PROVE), presented no significant effect on the perceived influence in work environments (*p* > 0.05) (Table 1).

#### 3.1.2. PROVE and the Perceived Influence in Policy Work Decisions

PROVE farmers present higher average scores for the perception of influence over policy decisions in the work environment when compared to the non-PROVE sample (mean = 6.74, standard deviation = 3.97*, n* = 35; mean = 5.37, standard deviation = 4.05*, n* = 36, respectively). 

From the selected controls, only gender presented a significant effect: females scored relatively higher than males on the perceived influence in work environments. Controlling for the effects of the age group, education group, gender, and economic difficulties, the sample effect (PROVE versus non-PROVE) attained statistical significance and thus indicated relevant differences between the PROVE and non-PROVE samples. Being a PROVE farmer correlates with higher scores for perceived influence in policy decisions related to the work organization‘s activities; on average, PROVE farmers scored almost three points higher on the scale (B = 1.104, *p* = 0.009) than the non-PROVE sample (Table 2).

Notes: Coefficients in non-standardized (B) and standardized (β) formats, and respective standard errors (BE), and interval confidence limits (95% level of confidence). Reference categories for the dummy variables for gender, age, education, and perceived economic difficulties between parentheses. * *p* < 0.05, *** *p* < 0.001.

### 3.2. PROVE and the Promotion of Healthier and More Sustainable Diets

#### 3.2.1. PROVE and the Intake of Fruits and Vegetables 

Around 39% of the study participants (*n* = 865) consumed at least five portions of fruits and vegetables per day. The regression models state that the likelihood rises significantly among PROVE consumers: membership of PROVE on average triples the chances of eating five portions a day of fruits and vegetables (odds ratio (OR) = 3.055, confidence interval (CI) = 2.08–4.48), after controlling for the effects of age, education, gender, partnership, and perceived economic difficulties (Table 3).

#### 3.2.2. PROVE and Red Meat Intake

Overall, 20% of participants (PROVE and non-PROVE) declared eating less than two portions (200 g) of red meat a week. Logistical regression coefficients on the chances of no more than two portions of red meat per week feature in Table 4. Once again, this identified significant differences between the samples: being a PROVE consumer raises the likelihood of eating no more than two portions of red meat a week (200 g) by about 55% (OR = 1.567, CI = 1.002–2.451), irrespective of age, education, gender, partnership, and economic difficulties.

## 4. Discussion

This article presents the key results of the evaluation of PROVE, a project that promotes the direct commercialization of seasonal fruits and vegetables from farmers to consumers. The evaluation study applies the intersectoral perspective proposed by the INHERIT model, studying the feasible implications of PROVE for fostering equity, health, and environmental sustainability. Building upon research on short food supply chains, PROVE was assessed regarding its possible influences over the empowerment of small-scale farmers and promoting healthier and more sustainable diet options among its consumers.

In line with previous research [9,10,11,12,13,14,15,16,17], the farmers involved in the PROVE short food supply chain reported relatively higher empowerment scores than the matched sample in one of the selected measures, returning higher scores for the perceived influence over policy decisions related to the activities of their work organization. 

The farmer empowerment generated by such programs interrelates with fostering access to resources, building their skills, and ensuring their voice and representation, alongside building a sense of identity and social value [11]. The set of tools, training, and infrastructure provided by PROVE theoretically interlinks with the intrapersonal dimensions related with empowerment, such as leadership, competence, and policy control [41]; influence and agency [42]; and operational capability [43]. 

The participant testimonies collected within the scope of the evaluation study corroborate these findings. In discussing PROVE’s impact on farmers, stakeholders explained how the PROVE program assists farmers in establishing local businesses and securing higher incomes based on network collaboration, planning, direct selling, and fair prices [33].PROVE farmers reported no advantages in terms of the perceived influence in daily work routines but higher scores for the perceived influence over decision-making in the working environment. Farming is a very labor-intensive activity requiring strict daily routines for agricultural tasks, especially when there are only low levels of mechanization as is common among small-scale farmers. The finding that PROVE farmers provide higher scores for their perceived influence on policy in the working environment is in line with expectations as we may expect network collaboration among farmers to influence planning and decision-making procedures and not their daily routines.

The comparative study of PROVE consumers correlates their participation in the program with a greater likelihood of not only eating the recommended amounts of fruits and vegetables for a healthy diet but also not exceeding the level of consumption of red meat required for sustainable diet patterns. Other programs to promote short chains for food supply have approached consumer motivations and dietary behaviors (e.g., [10,27,28,29]). The opinions collected from among PROVE stakeholders regarding the program’s impact highlighted the project’s role in changing diets. According to the perceptions collected, PROVE consumers are more able to introduce fruits and vegetables into household meals throughout the week due to the program; reporting not only an increase in quantity but also an increase in the variety of fruits and vegetables consumed [33]. It is possible that consumers adhering to programs such as PROVE may already have increased levels of motivation and knowledge in relation to healthier diets. Nonetheless, theoretically we may plausibly assume that participating in such programs can also positively influence key determinants for dietary change. Participating in these programs can hold important benefits, such as to reinforce knowledge, positive beliefs, and attitudes toward healthier and more sustainable dietary options [44]; influence intentional and non-intentional triggers to eat fruits and vegetables [45]; and increase opportunities for choosing healthier and more sustainable meals [46,47,48]. 

This evaluation incorporated a set of comparative studies between PROVE users and samples sourced from national representative surveys. Within the program evaluation research framework, this adopts the post-test-only non-equivalent group design option. From a methodological perspective, we believe that this study offers a valid perspective for the evaluation of real-life ongoing small circuit projects and may be a valid example for other similar cases. However, this strategy also holds important limitations. 

As participants are not randomly assigned to one or the other group, there may be a threat to the validity of the study due to selection bias [34]. Certain strategies were put into place to address selection bias and sample comparability. In data collection for the farmers study, all the listed contacts were used in the national participant recruitment process, and data collection was conducted by telephone in order to facilitate participation and convenience. Additionally, PROVE consumer recruitment received the support of an online campaign that applied a similar study description to that of the INHERIT Five-Country Survey. In the data analysis, to ensure differences in the outcome variables are not confounded by differences in socioeconomic compositions, PROVE users were compared with matched subsamples (selected by matching propensity procedures) and with the inclusion of relevant socioeconomic indicators in the regression models as covariates. Nevertheless, ensuring similarities between PROVE users and non-users was difficult to achieve in this design, especially in the case of the farmers study given farmers are subject to comparison with people in other occupations (European Social Survey did not present enough cases to restrict the analysis only to other farmers). This brings important difficulties to the study and for the extensions of the observed findings. Additionally, the program may be aggregating particularly empowered farmers and consumers oriented by health and sustainability concerns. If this was the case, differences between PROVE and non-PROVE samples can be referring to previous differences rather than changes promoted by the program. Hence, any generalization of these results still requires certain reservations. 

The strategy also obliged restricting the evaluation focus to indicators that featured in data sets available to the research team. Access to the INHERIT Five-Country Survey ensured high quality and comparative data on fruits, vegetables, and meat intakes, based on a validated instrument for collecting diet patterns [38]. However, the measurement of empowerment is less well expressed given it spans only the perceptions on personal influence in workplace settings. Other measures might better assess the implications for farmer empowerment, such as the empowerment dynamics index developed to map the implications of a farming project program for empowerment across multiple spheres—specifically, the economic, family, political, knowledge, and psychological dimensions [49].

Future research should address the consistency of these results in more controlled circumstances, for example applying randomized controlled trials or stepped-wedged designs. It would be also relevant to explore the pathways by which PROVE influences the empowerment of farmers and the diets of consumers. Additionally, other dimensions of health, sustainability, and equity might be addressed in future intersectoral evaluations. For example, studies might examine the health benefits of PROVE consumers deriving from diet diversity, the sustainability benefits related to farming practices, and the relative savings from food distribution (lower food miles). Also, studies could explore PROVE contributions to reducing dietary inequalities, comparing their effects on higher and lower socioeconomic families and the implementation strategies for reaching out to lower socioeconomic groups. 

Despite the aforementioned limitations, results from this study indicate that PROVE farmers felt more empowered in their ability to influence policy decisions regarding activities of the organization or their PROVE group. Moreover, being a PROVE consumer was also associated with greater intake of fruits and vegetables and lower consumption of red meat, which are two important aspects of a healthy and sustainable dietary pattern. Hence, PROVE is an effective practice in fostering behaviors that contribute to health, sustainability, and equity. It can also be considered a good practice [50] in that it consists of an intervention that is implemented in a real-life setting that is adequate to its content and purpose, well-accepted by its consumers and stakeholders, ethical and fair, and that contributes to equity and important health-relevant outcomes. Importantly, this model of short distance supply chain is sustainable and replicable in that it provides a full toolkit, including a handbook with a detailed description of all methodologies, and an online platform allowing producers to connect with consumers. In this sense, it shows high transferability potential to other regions and countries.

## 5. Conclusions

PROVE is a national program that provides tools, training, and partnerships to enable small-scale farmers organized into local networks to sell local seasonal fruits and vegetables. According to this research evaluation, the evidence suggests PROVE contributes to the empowerment of small-scale farmers and the consumption of fruits and vegetables among its consumers. 

These results promote the idea that such programs promote fair farming and facilitate the consumption of seasonal and local food. Scaling up this methodology for setting up short distance supply chains may contribute to a healthier, more sustainable, and fairer food system.

## Figures and Tables

**Table 1:** Regression coefficients on perceived influence in work environments—Influence in Daily Work *(n* = 69).

Variables	B	SE	Exp(B)	95% CI Limits	
				Lower	Upper
Gender: female (male)	0.360	0.494	1.433	0.5441	3.7730
Age: 18–34 years (50+ years)	0.217	0.822	1.243	0.2479	6.2293
Age: 35–49 years (50+ years)	−0.437	0.527	0.646	0.2298	1.8144
Education: primary/lower secondary (tertiary)	−1.315 *	0.549	0.269	0.0916	0.7869
Education: upper secondary (tertiary)	−1.021	0.691	0.360	0.0928	1.3980
No economic difficulties (economic difficulties)	−0.477	0.627	0.620	0.1815	2.1209
PROVE	−0.009	0.450	0.991	0.4101	2.3964

Notes: Non-standardized coefficients (B), odds ratio (Exp(B)) and respective confidence interval limits (95% level of confidence). Reference categories for the dummy variables for gender, age, education, and perceived economic difficulties between parentheses. * *p* < 0.05.

**Table 2:** Regression coefficients on perceived influence in work environments – Influence in Policy Decisions *(n* = 69).

Variables	B	SE	Exp(B)	95% CI Limits	
				Lower	Upper
Gender: female (male)	1.043 *	0.493	2.838	1.080	7.457
Age: 18–34 years (50+ years)	0.264	0.844	1.302	0.249	6.801
Age: 35–49 years (50+ years)	−0.185	0.503	0.831	0.310	2.226
Education: primary/lower secondary (tertiary)	−0.830	0.531	0.436	0.154	1.236
Education: upper secondary (tertiary)	−0.244	0.652	0.783	0.218	2.813
No economic difficulties (economic difficulties)	−0.485	0.618	0.616	0.183	2.069
PROVE	1.104 ***	0.459	3.016	1.228	7.411

Notes: Coefficients in non-standardized (B) and standardized (β) formats, and respective standard errors (BE), and interval confidence limits (95% level of confidence). Reference categories for the dummy variables for gender, age, education, and perceived economic difficulties between parentheses. * *p* < 0.05, *** *p* < 0.001.

**Table 3:** Regression coefficients for the likelihood of eating five portions a day of fruits and vegetables (*n* = 840).

Variables	B	SE	Exp(B)	95% CI Limits	
				Lower	Upper
Gender: female (male)	−0.655 ***	0.181	0.519	0.365	0.740
Gender: no information (male)	−0.245	0.337	0.783	0.404	1.517
Age: 18–34 years (50+ years)	−0.992 ***	0.245	0.371	0.229	0.600
Age: 35–49 years (50+ years)	−0.827 ***	0.196	0.437	0.298	0.642
Education: primary/lower secondary (tertiary)	−1.201 ***	0.306	0.301	0.165	0.548
Education: upper secondary (tertiary)	−0.624 ***	0.214	0.536	0.352	0.815
No economic difficulties (economic difficulties)	0.355 *	0.178	1.426	1.007	2.021
Partner (no partner)	0.156	0.175	1.169	0.829	1.647
PROVE	1.117 ***	0.196	3.055	2.082	4.484

Notes: Non-standardized coefficients (B), odds ratio (Exp(B)) and respective confidence interval limits (95% level of confidence). Reference categories for the dummy variables for gender, age, education, perceived economic difficulties, and partner between parentheses. * *p* < 0.05, *** *p* < 0.001

**Table 4:** Regression coefficients for the likelihood of eating less than two portions of meat a week (*n* = 840).

Variables	B	SE	Exp(B)	95% CI Limits	
				Lower	Upper
Gender: female (male)	−0.716 ***	0.227	0.489	0.313	0.763
Gender: no information (male)	−0.757	0.442	0.469	0.197	1.115
Age: 18–34 years (50+ years)	0.159	0.280	1.173	0.678	2.030
Age: 35–49 years (50+ years)	−0.164	0.230	0.849	0.541	1.334
Education: primary/lower secondary (tertiary)	0.048	0.342	1.049	0.537	2.049
Education: upper secondary (tertiary)	−0.047	0.253	0.954	0.581	1.566
No economic difficulties (economic difficulties)	0.044	0.211	1.045	0.691	1.580
Partner (no partner)	−0.111	0.203	0.895	0.602	1.332
PROVE	0.449 *	0.228	1.567	1.002	2.451

Notes: Non-standardized coefficients (B), odds ratio (Exp(B)) and respective confidence interval limits (95% level of confidence). Reference categories for the dummy variables for gender, age, education, perceived economic difficulties, and partner between parentheses. * *p* < 0.05, *** *p* < 0.001.

## Supplementary Materials

The following are available online at https://www.mdpi.com/1660-4601/16/24/5083/s1.
