# Supplementary material for: Equity, Health, and Sustainability with PROVE: The Evaluation of a Portuguese Program for a Short Distance Supply Chain of Fruits and Vegetables
Source: Int J Environ Res Public Health. 2019 Dec 12;16(24):5083. doi: 10.3390/ijerph16245083 (PMC6950233; doi:10.3390/ijerph16245083)
Supplement: Supplementary file 1 [file ijerph-16-05083-s001.pdf]

Supplementary Files

1. Bias treatment-effects test for the farmers' studies

Table A1 shows the results of the bias treatment approaches for adjustment of potential heterogeneity in the sample. The estimations show that farmers who actually participated in the PROVE project does not show significant differences with respect to individulas that did not participate, and so not being observed endogeneity problems among both decision stages (i.e., treatment and no-treatment scenarios). It can be observed that **ϱ** is not significant in Heckman. It is not possible to estimate an ESR model in this case for conformability matrix problems due to different number of scores in treatment and no-treatment scenarios. Therefore, unobserved heterogeneity seems not to be a problem here and Logit or Probit models not presenting sample selection bias problems in this case.

Table A1. Bias treatment-effects test for the farmers' studies

**Infuence in daily work**				
	**Treatment B (s.e.)**	**IC95% limits**	**ϱ (Treatment)**	**LR**
Logit model	-.009 (,450)	(-.891, .874)		
Probit model	.040 (.265)	(-.478, .560)		
Heckman model[1]	.041 (.265)	(-.479, .560)	.029 (1.420)	$\chi^2(1)$ =0.00
**Infuence in policy decisions**				
	**Treatment B (s.e.)**	**IC95% limits**	**ϱ (Treatment)**	**LR**
Logit model	1.104 (.459)***	(.205, 2.003)		
Probit model	.668 (.2657)***	(.1474, 1.189)		
Heckman model[12]	.668 (.265)***	(.147, 1.189)	.004 (2.162)	$\chi^2(1)$ =0.00

[1] Full information maximum likelihood estimates. [2] Education variables were dropped from selection equation due to collinearity problems.

2. Bias treatment-effects test for the consumers studies

Table A2 shows the results of the ESR approaches for adjustment of potential heterogeneity in the sample. The estimations show that individuals who actually participated in the PROVE project does not show significant differences with respect to individuals that did not participate, and so not being observed endogeneity problems among both decision stages (i.e., treatment and no-treatment scenarios). It can be observed that **ϱ** is not significant neither in Heckman nor in ESR model estimates. Therefore, unobserved heterogeneity seems not to be a problem here and Logit and Probit models not presenting sample selection bias problems in this case.

Table A2. Bias treatment-effects test for consumers studies

Fruits and vegetables (5 portions a day)

	Treatment B (s.e.)	IC95% limits	ϱ (Treatment)	ϱ (Not treatment)	LR
Logit model	1.117 (.196)***	(.733, 1.501)			
Probit model	.683 (.119)***	(.450, .916)			
Heckman model[1]	.641 (.197)***	(.254, 1.027)	-.074 (.2709311)		$\chi^2(1) = 0.07$
Endogenous switching regression model[1]	.641 (.197)***	(.254, 1.027)	-.074 (.2709259)	-.004 (.144)	$\chi^2(2) = 0.07$

Red meat (less than 2 portions a week)

	Treatment B (s.e.)	IC95% limits	ϱ (Treatment)	ϱ (Not treatment)	LR
Logit model	.449 (.228)**	(.002, .897)			
Probit model	.266 (.131)**	(.009, .523)			
Heckman model[1]	.086 (.226)	(-.358, .531)	-.279 (.262)		$\chi^2(1) = 0.98$
Endogenous switching regression model[1, 2]	.087 (.225)	(-.354, .529)	-.283 (.264)	.121 (.081)	$\chi^2(2) = 3.20$

[1] Full information maximum likelihood estimates. [2] Education variables were dropped from not treatment equation due to collinearity problems.
